# Supplementary material for: Viral infection of cells within the tumor microenvironment mediates antitumor immunotherapy via selective TBK1-IRF3 signaling
Source: Nat Commun. 2021 Mar 25;12:1858. doi: 10.1038/s41467-021-22088-1 (PMC7994570; doi:10.1038/s41467-021-22088-1)
Supplement: Supplementary file 1 — Supplementary Information [file 41467_2021_22088_MOESM1_ESM.pdf]

Supplementary Information

Viral Infection of the Tumor Microenvironment Mediates Antitumor Immunotherapy via Selective TBK1-IRF3 Signaling

Brown *et al.*

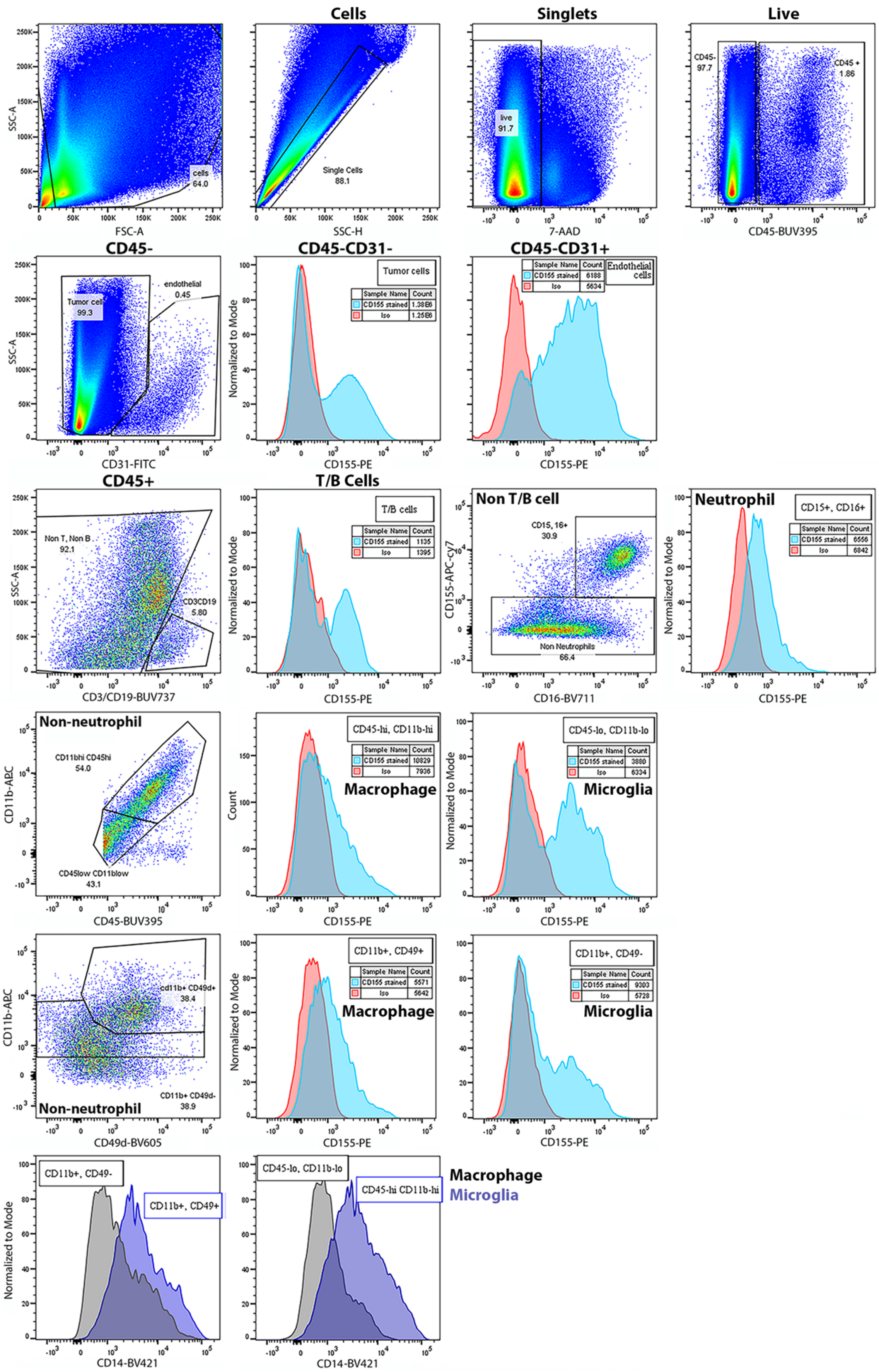

Cells
Singlets
Live
FSC-A
SSC-A
cells 64.0
SSC-H
Single Cells 88.1
7-AAD
live 91.7
CD45-BUV395
CD45- 97.7
CD45 + 1.86
CD45-
CD45-CD31-
CD45-CD31+
CD31-FITC
Tumor cell 99.3
endothelial 0.45
Tumor cells
Endothelial cells
Sample Name
Count
CD155 stained
Iso
Normalized to Mode
CD155-PE
CD45+
T/B Cells
Non T/B cell
Neutrophil
CD3/CD19-BUV737
Non T, Non B 92.1
CD3CD19 5.80
T/B cells
CD155-APC-cy7
CD16-BV711
CD15, 16+ 30.9
Non Neutrophils 66.4
CD15+, CD16+
Non-neutrophil
CD11b-APC
CD45-BUV395
CD11bhi CD45hi 54.0
CD45low CD11blow 43.1
CD45-hi, CD11b-hi
CD45-lo, CD11b-lo
Macrophage
Microglia
Count
CD49d-BV605
cd11b+ CD49d+ 38.4
CD11b+ CD49d- 38.9
CD11b+, CD49+
CD11b+, CD49-
CD14-BV421
CD45-hi CD11b-hi

Supplementary Figure 1, related to Figure 1b. Representative gating strategy for analysis of CD155 expression distribution in GBM tumor specimens.

Red histograms are fluorescence minus one isotype control-PE antibody stained, light blue histograms are CD155-PE stained. Bottom 2 panels show comparison of CD14 expression in microglia and macrophages.

GBM tissue-matched cytokine induction

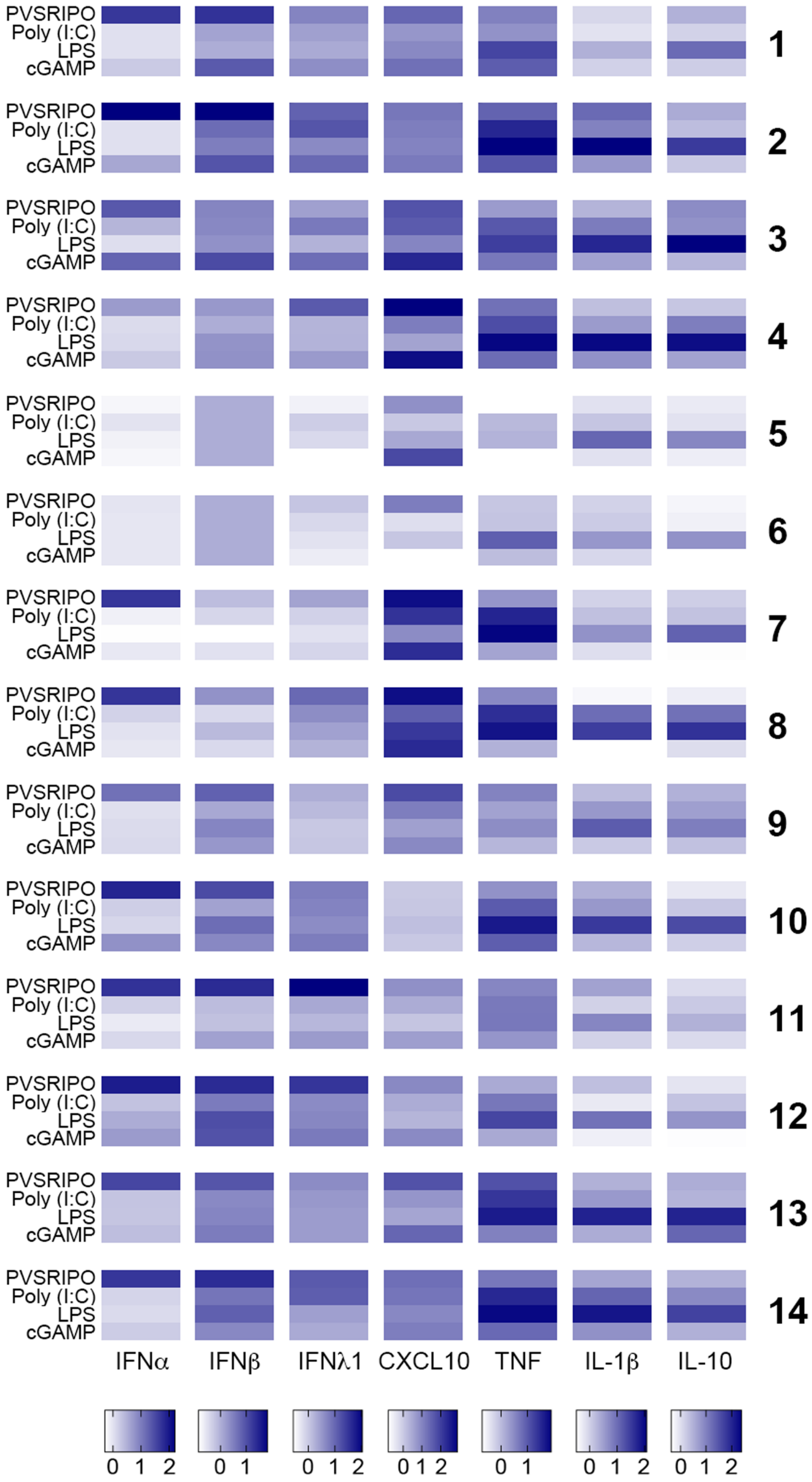

Supplementary Figure 2, related to Figure 1d. Patient-specific heatmaps depicting fold-change induction from mock control for each cytokine.

Only patients for which all 4 treatments were tested are shown (n=14); concentrations and experimental design are described in Figure 1.

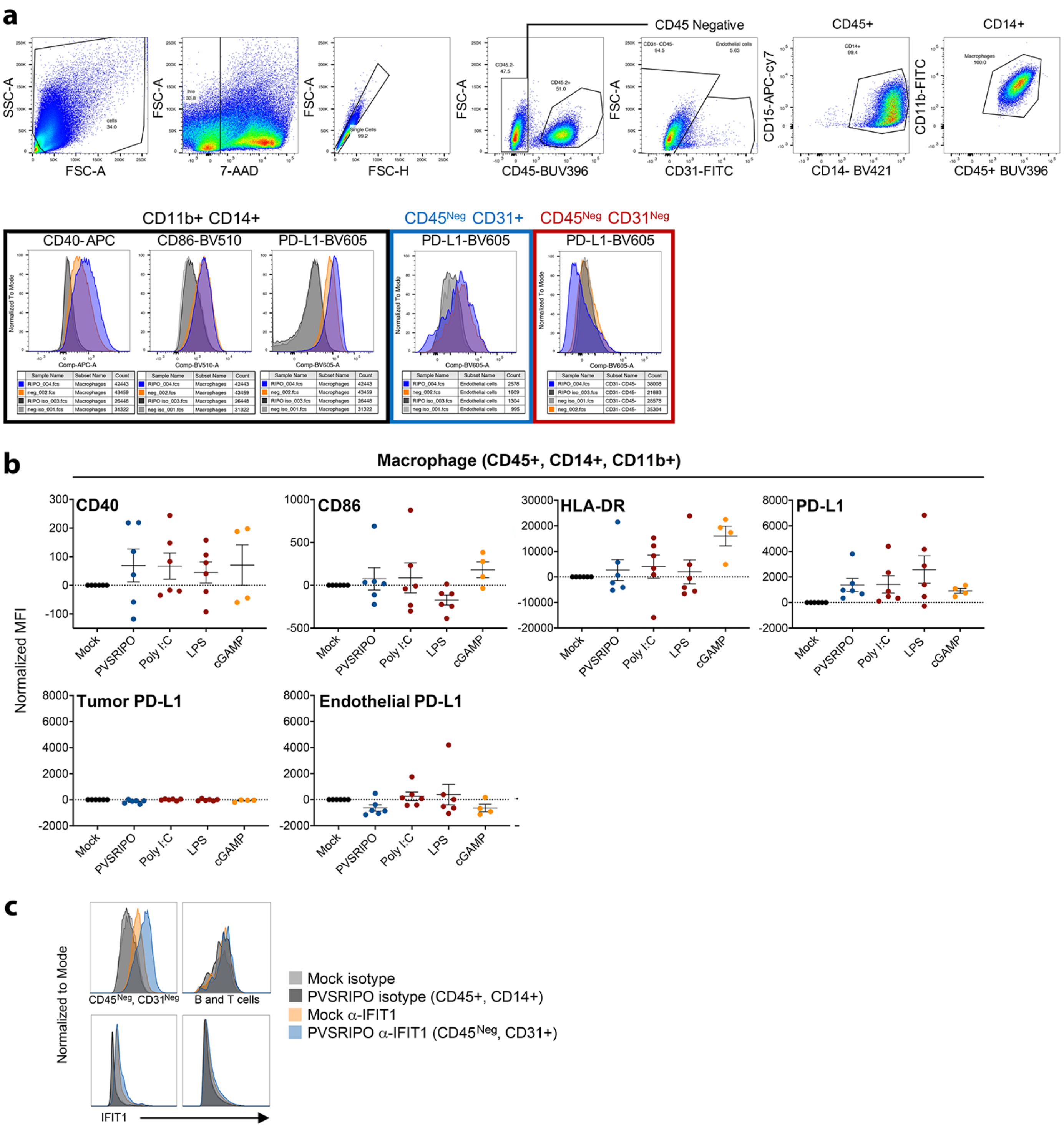
a
SSC-A
FSC-A
FSC-A
7-AAD
FSC-A
FSC-H
FSC-A
CD45-BUV396
CD45 Negative
FSC-A
CD31-FITC
CD45+
CD15-APC-cy7
CD14- BV421
CD14+
CD11b-FITC
CD45+ BUV396
CD11b+ CD14+
CD40-APC
CD86-BV510
PD-L1-BV605
CD45^Neg CD31+
PD-L1-BV605
CD45^Neg CD31^Neg
PD-L1-BV605
b
Macrophage (CD45+, CD14+, CD11b+)
Normalized MFI
CD40
CD86
HLA-DR
PD-L1
Tumor PD-L1
Endothelial PD-L1
Mock
PVSRIPO
Poly I:C
LPS
cGAMP
c
Normalized to Mode
CD45^Neg, CD31^Neg
B and T cells
IFIT1
Mock isotype
PVSRIPO isotype (CD45+, CD14+)
Mock α-IFIT1
PVSRIPO α-IFIT1 (CD45^Neg, CD31+)

Supplementary Figure S3, related to Figure 1f and g. Gating strategy for tumor-associated macrophage activation and IFIT1 status after in vitro slice culture assay.

(**a**) Representative gating strategy for macrophage activation status in GBM and breast cancer specimens after *ex vivo* treatment (n=6 GBM, n= 2 breast cancer were tested); MFI (median florescence intensity) is shown on the x axis for histograms (bottom). (**b**) Post-treatment slice culture-derived tumor associated macrophages, endothelial cells, and tumor cells analyzed for PD-L1 in Figure 1f were also tested for CD40, CD86, HLA-DR; individual values are shown for each patient specimen and data brackets indicate mean -/+ SEM (n=6 GBM). (**c**) Representative histograms for intracellular IFIT1 staining after gating performed as in (a); data from all tested samples (n=4 GBM, n=2 breast cancer) presented in Fig. 1g.

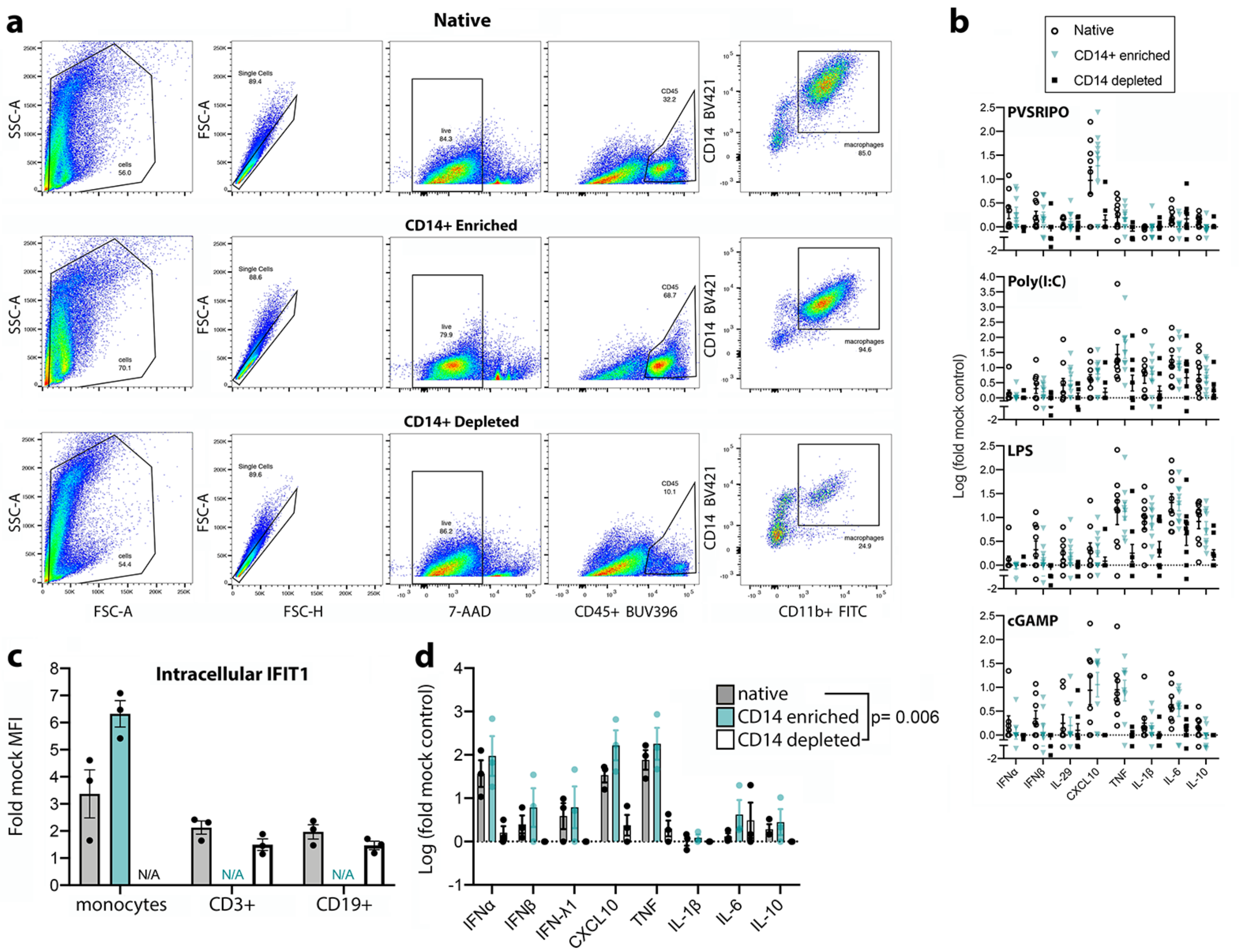
a
Native
CD14+ Enriched
CD14+ Depleted
SSC-A
FSC-A
FSC-H
7-AAD
CD45+ BUV396
CD14 BV421
CD11b+ FITC
b
Native
CD14+ enriched
CD14 depleted
PVSRIPO
Poly(I:C)
LPS
cGAMP
Log (fold mock control)
IFNα
IFNβ
IL-29
CXCL10
TNF
IL-1β
IL-6
IL-10
c
Intracellular IFIT1
Fold mock MFI
N/A
monocytes
CD3+
CD19+
d
native
CD14 enriched
CD14 depleted
p= 0.006
Log (fold mock control)
IFNα
IFNβ
IFN-λ1
CXCL10
TNF
IL-1β
IL-6
IL-10

Supplementary Figure S4, related to Figure 1g and h. Cytokine responses of tumor cell suspensions and PBMCs after PVSRIPO/PRR agonist treatment depend upon CD14+ myeloid cells.

(**a**) Representative data assessing CD14+ cell enrichment/depletion for GBM single cell suspensions. (**b**) Individual data points for cytokine data presented in Figure 1h, including cytokine secretion from CD14+ cells (from n= 9 tumors). (**c**, **d**) Human PBMCs (n= 3 donors) were treated with mock or PVSRIPO after CD14+ pre-depletion, post-depletion, or CD14+ purified fractions, and tested for IFIT1 staining (**c**) as in Figure 1g, or supernatant cytokine analysis (**d**) as in Figure 1h; p-value is from paired t test (two-tailed) comparing sum fold-mock induction. (**b-d**) All data bars/brackets indicate mean -/+ SEM.

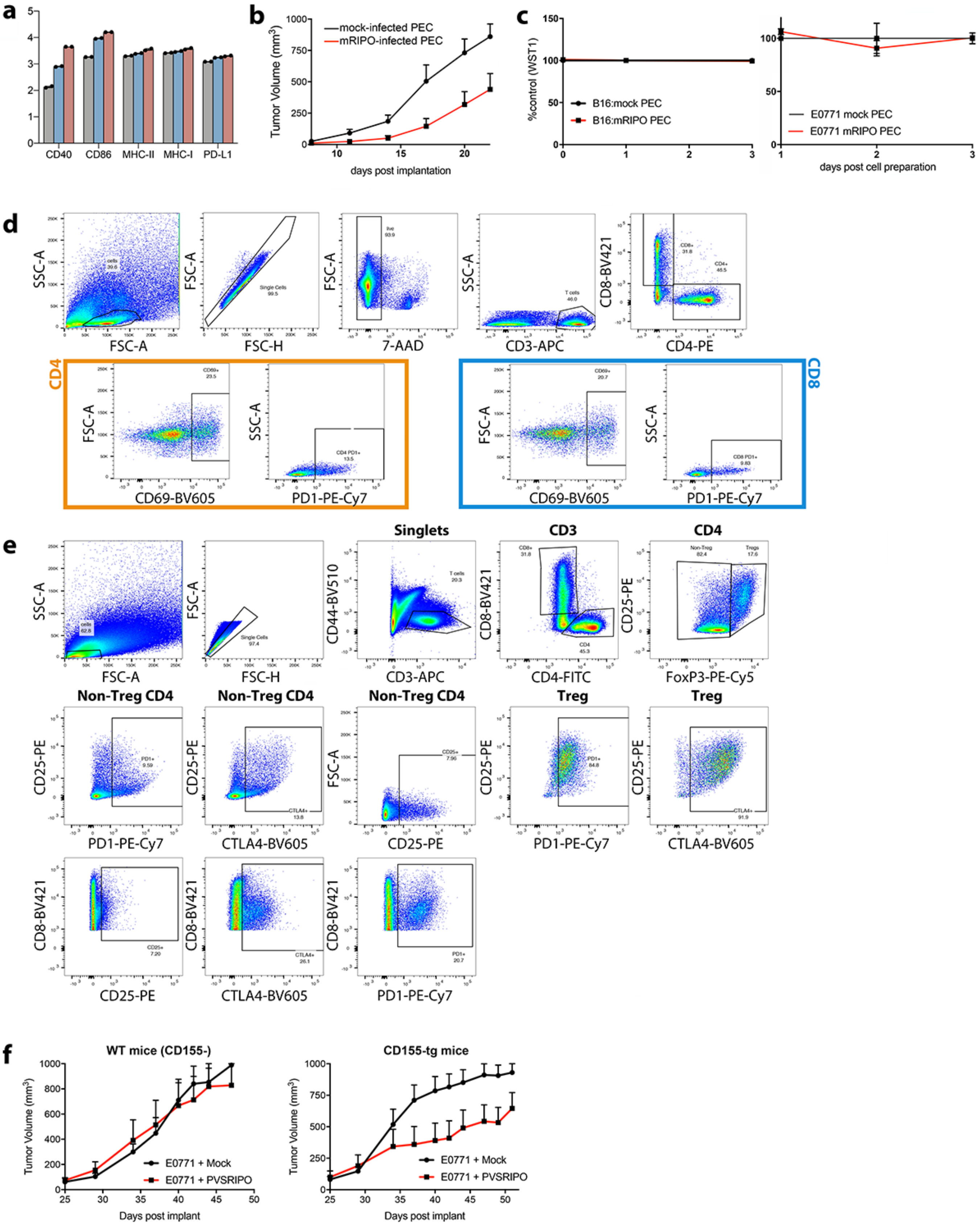

a
CD40
CD86
MHC-II
MHC-I
PD-L1
b
Tumor Volume (mm3)
mock-infected PEC
mRIPO-infected PEC
days post implantation
c
%control (WST1)
B16:mock PEC
B16:mRIPO PEC
E0771 mock PEC
E0771 mRIPO PEC
days post cell preparation
d
SSC-A
FSC-A
FSC-H
7-AAD
CD3-APC
CD8-BV421
CD4-PE
CD4
CD8
CD69-BV605
PD1-PE-Cy7
e
Singlets
CD3
CD4
CD44-BV510
CD4-FITC
CD25-PE
FoxP3-PE-Cy5
Non-Treg CD4
Treg
CTLA4-BV605
f
WT mice (CD155-)
CD155-tg mice
Tumor Volume (mm3)
E0771 + Mock
E0771 + PVSRIPO
Days post implant

Supplementary Figure 5, related to Figures 2 and 3. Murine myeloid cells and the non-malignant TME mediate antitumor efficacy in response to mRIPO infection.

(**a**) Flow cytometry analysis of PEC treated with Mock (gray bars), mRIPO (MOI 10, blue bars) or LPS (100ng/ml, red bars) (24h), expressed as log MFI for each cell surface marker (n=2 experiments). (**b**) Repeat experiment for series presented in Figure 2b, left panel (B16, n= 10 mice/group); mean tumor volume + SEM is shown. (**c**) Tumor cell preparations used for implantation into mice in Figure 2b were plated in 96 well plates (1000 cells per well) and analyzed by WST1 assay for viability for 3 days; B16 (left panel) and E0771 (right panel), a representative series is shown from n=2 cell preparations per model; mean -/+ SEM is plotted from technical replicates within one experiment. (**d**) Representative gating strategy for T cell panel testing CD69 and PD1. (**e**) Representative gating strategy used to determine Treg and non-Treg CD4 phenotypes in Figure 2c. (**f**) WT E0771 cells were co-implanted into the fat pad of WT (left panel) or CD155-tg (right panel) mice with or without $1x10^8$ pfu mRIPO; mean tumor volume and SEM is shown (left panel: n=4/group; right panel: n=10/group).

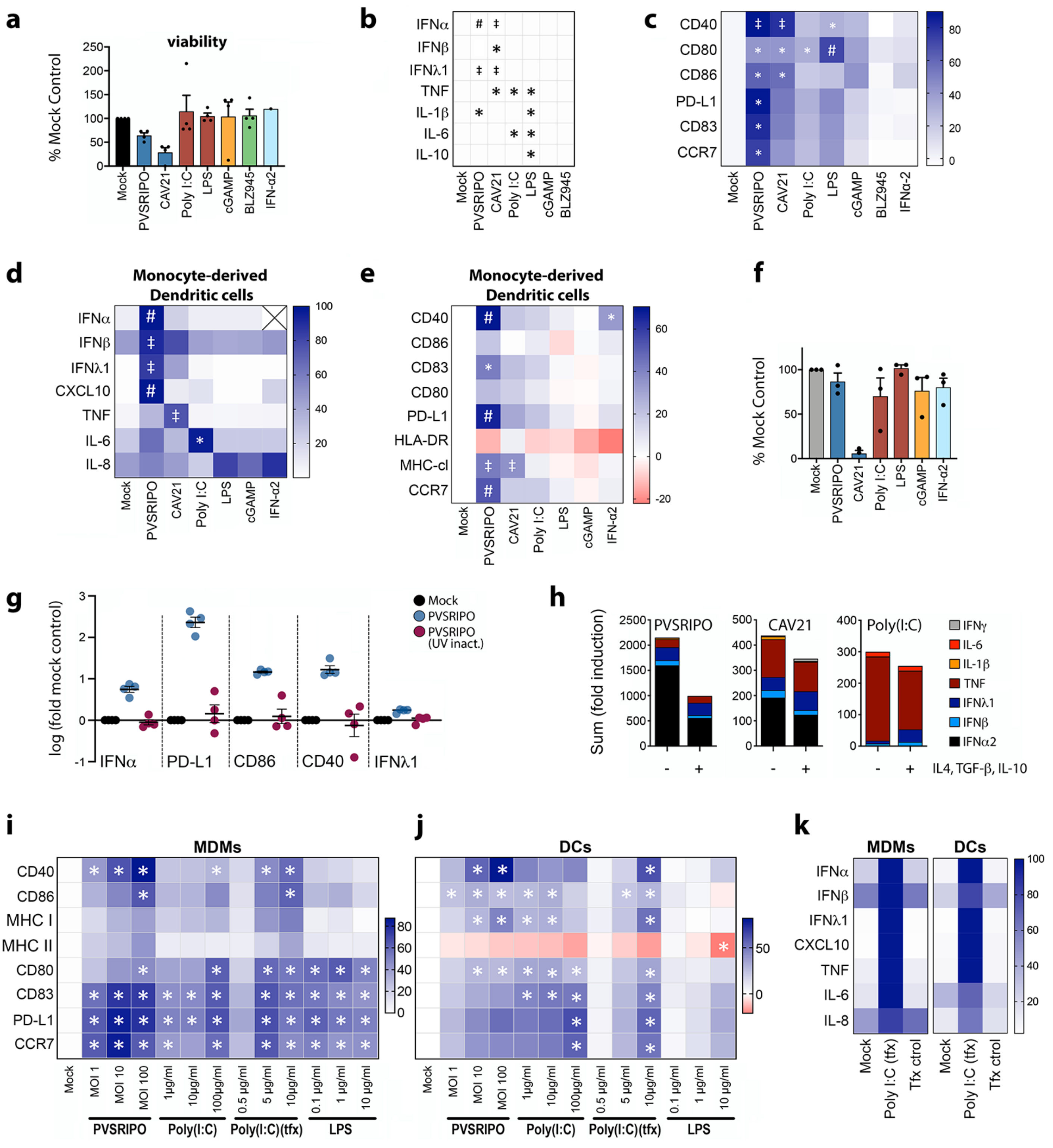
a
viability
% Mock Control
250
200
150
100
50
0
Mock
PVSRIPO
CAV21
Poly I:C
LPS
cGAMP
BLZ945
IFN-α2
b
IFNα
IFNβ
IFNλ1
TNF
IL-1β
IL-6
IL-10
Mock
PVSRIPO
CAV21
Poly I:C
LPS
cGAMP
BLZ945
c
CD40
CD80
CD86
PD-L1
CD83
CCR7
Mock
PVSRIPO
CAV21
Poly I:C
LPS
cGAMP
BLZ945
IFNα-2
80
60
40
20
0
d
Monocyte-derived
Dendritic cells
IFNα
IFNβ
IFNλ1
CXCL10
TNF
IL-6
IL-8
Mock
PVSRIPO
CAV21
Poly I:C
LPS
cGAMP
IFN-α2
100
80
60
40
20
e
Monocyte-derived
Dendritic cells
CD40
CD86
CD83
CD80
PD-L1
HLA-DR
MHC-cl
CCR7
Mock
PVSRIPO
CAV21
Poly I:C
LPS
cGAMP
IFN-α2
60
40
20
0
-20
f
% Mock Control
100
50
0
Mock
PVSRIPO
CAV21
Poly I:C
LPS
cGAMP
IFN-α2
g
log (fold mock control)
3
2
1
0
-1
IFNα
PD-L1
CD86
CD40
IFNλ1
Mock
PVSRIPO
PVSRIPO (UV inact.)
h
Sum (fold induction)
PVSRIPO
2500
2000
1500
1000
500
0
CAV21
500
400
300
200
100
0
Poly(I:C)
400
300
200
100
0
IFNγ
IL-6
IL-1β
TNF
IFNλ1
IFNβ
IFNα2
-
+
IL4, TGF-β, IL-10
i
MDMs
CD40
CD86
MHC I
MHC II
CD80
CD83
PD-L1
CCR7
80
60
40
20
0
Mock
MOI 1
MOI 10
MOI 100
1μg/ml
10μg/ml
100μg/ml
0.5 μg/ml
5 μg/ml
10μg/ml
0.1 μg/ml
1 μg/ml
10 μg/ml
PVSRIPO
Poly(I:C)
Poly(I:C)(tfx)
LPS
j
DCs
50
0
Mock
MOI 1
MOI 10
MOI 100
1μg/ml
10μg/ml
100μg/ml
0.5 μg/ml
5 μg/ml
10μg/ml
0.1 μg/ml
1 μg/ml
10 μg/ml
PVSRIPO
Poly(I:C)
Poly(I:C)(tfx)
LPS
k
MDMs
DCs
IFNα
IFNβ
IFNλ1
CXCL10
TNF
IL-6
IL-8
Mock
Poly I:C (tfx)
Tfx ctrol
100
80
60
40
20

Supplementary Figure 6, related to Figure 4. Enteroviral replication/cytoplasmic dsRNA induce a distinct type-I/III IFN dominant phenotype in human macrophages and DCs.

(**a**) Viability determined by WST1 assay for MDMs after various treatments as shown in Figure 4a (n=4 experiments, except IFNα2 which was tested in this assay once); mean + SEM is shown. (**b**) Tukey's post hoc test p<0.05 (two-tailed) comparing each individual treatment in Figure 4a to mock (*), all other groups except PVSRIPO or CAV21 (‡), or (#) all other groups. (**c**) Flow cytometry analysis of activation markers on macrophages from Figure 4a (n=4 experiments/group except for cGAMP, BLZ945, and IFNα2 where n=2); symbols indicate same statistical information as in (b), comparisons were not performed for cGAMP, BLZ495, or IFNα2 due to n=2. (**d-f**) Cytokine (**d**), flow cytometry analysis (**e**), and viability (**f**, WST1 assay, data bars represent mean -/+ SEM) of monocyte-derived DCs treated as in Figure 4a (d and e n=4, f n=3 experiments); symbols indicate same as in (b). (**g**) MDMs were treated with Mock, PVSRIPO, or UV-inactivated PVSRIPO and supernatant was assayed for cytokine release. Cell surface staining (PD-L1, CD86, CD40) was also performed; relevant cytokines and cell surface markers are shown (n=4 experiments from 2 different donors) normalized between experiments by determining log-fold mock control. Brackets indicate mean values and SEM. (**h**) Macrophages were differentiated with M-CSF alone or in combination with IL-4, TGF-β, and IL-10 (all 20pg/ml) and treated with mock, PVSRIPO (MOI 10), CAV21 (MOI 10), or Poly(I:C) (10μg/ml). Cytokine release was measured and fold-mock control values were plotted together in each data bar to depict relative contribution to total cytokine induction (n=2 experiments). (**i**, **j**) Accompanying flow cytometry data for cells form experiments in Figure 4c (**i**) and 4d (**j**); %maximum MFI was determined followed by subtracting mock values for each activation marker; (*) indicates Tukey's post hoc test p<0.05 (two-tailed) vs mock treatment. (**k**) Comparison of Poly(I:C)-TFX vs transfection reagent (lyovec) alone in MDMs and DCs as indicated; %maximum concentration is shown.

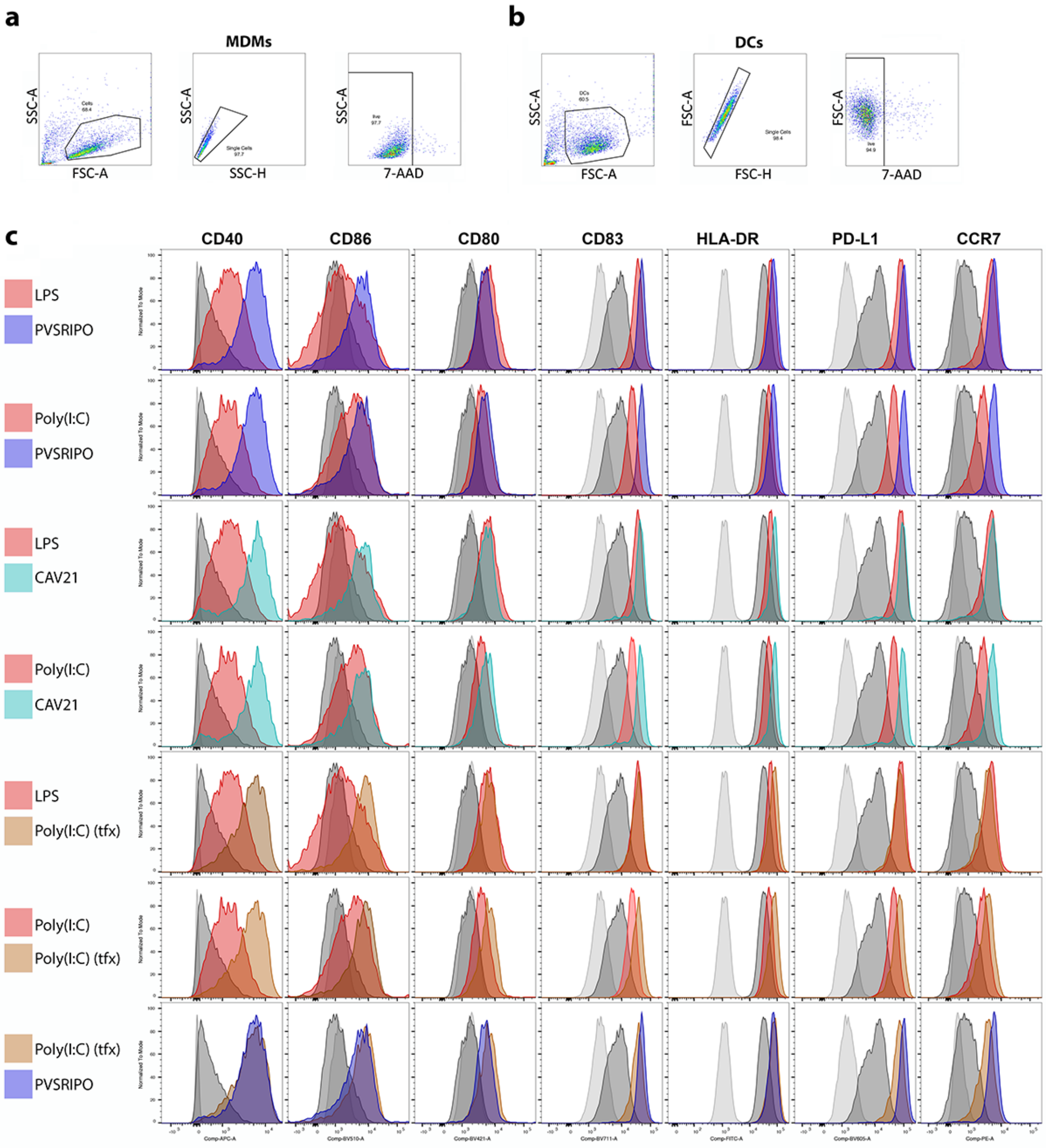

Supplementary Figure 7, related to Figure 4. Analysis of surface activation marker phenotype after enterovirus/cytoplasmic dsRNA compared to TLR agonists.

Gating strategy for MDMs (**a**) and DCs (**b**); histograms for surface activation markers on MDMs from a representative experiment (**c**).

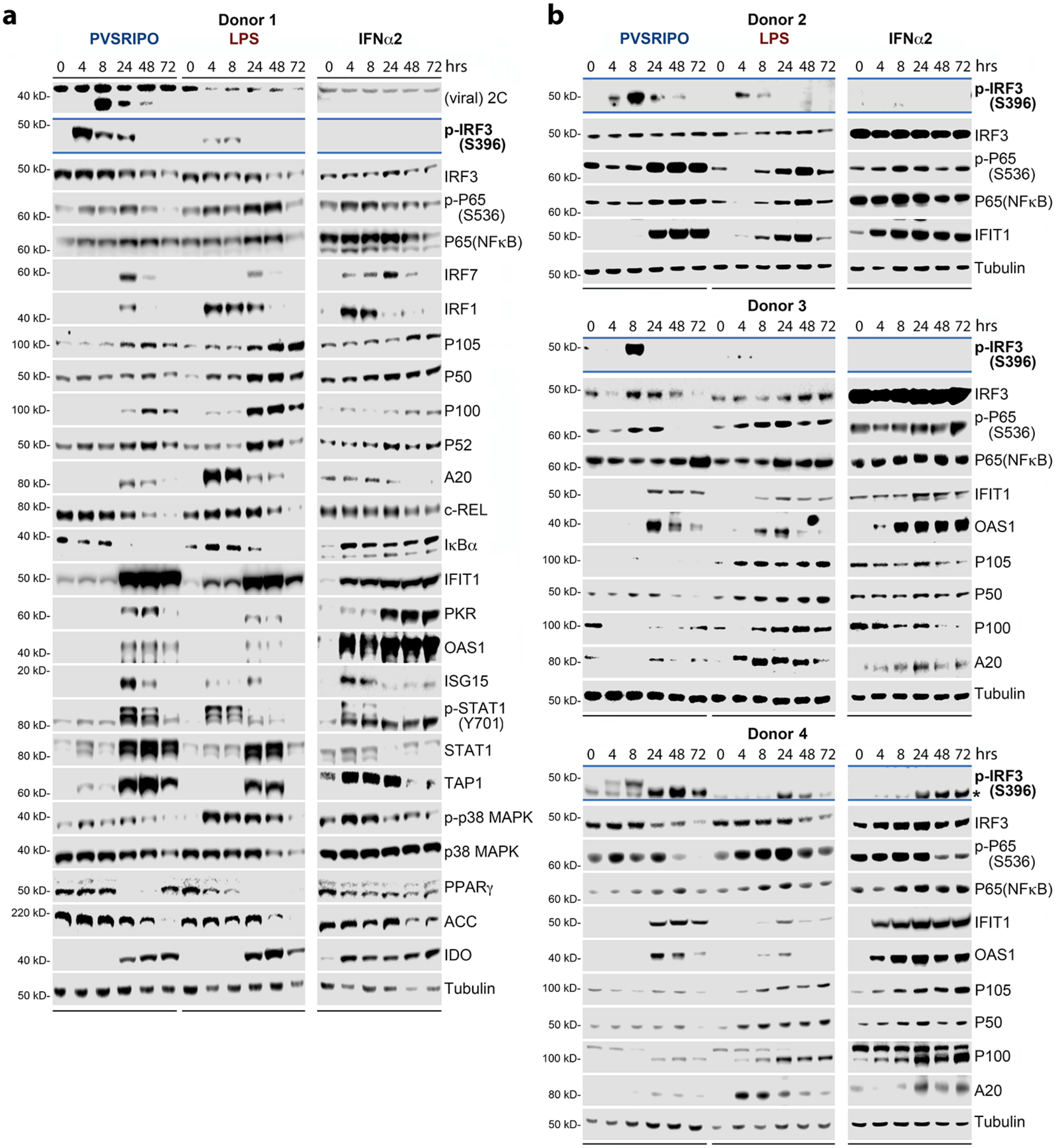
a
Donor 1
PVSRIPO
LPS
IFNα2
0 4 8 24 48 72 0 4 8 24 48 72 0 4 8 24 48 72 hrs
(viral) 2C
p-IRF3 (S396)
IRF3
p-P65 (S536)
P65(NFκB)
IRF7
IRF1
P105
P50
P100
P52
A20
c-REL
IκBα
IFIT1
PKR
OAS1
ISG15
p-STAT1 (Y701)
STAT1
TAP1
p-p38 MAPK
p38 MAPK
PPARγ
ACC
IDO
Tubulin
40 kD-
50 kD-
60 kD-
80 kD-
100 kD-
20 kD-
220 kD-
b
Donor 2
PVSRIPO
LPS
IFNα2
0 4 8 24 48 72 0 4 8 24 48 72 0 4 8 24 48 72 hrs
p-IRF3 (S396)
IRF3
p-P65 (S536)
P65(NFκB)
IFIT1
Tubulin
Donor 3
0 4 8 24 48 72 0 4 8 24 48 72 0 4 8 24 48 72 hrs
p-IRF3 (S396)
IRF3
p-P65 (S536)
P65(NFκB)
IFIT1
OAS1
P105
P50
P100
A20
Tubulin
Donor 4
0 4 8 24 48 72 0 4 8 24 48 72 0 4 8 24 48 72 hrs
p-IRF3 (S396)
*
IRF3
p-P65 (S536)
P65(NFκB)
IFIT1
OAS1
P105
P50
P100
A20
Tubulin

Supplementary Figure 8, related to Figure 5. **Immunoblots from 4 different donors, including all blots quantitated in Figure 5b.**

Each immunoblot series was performed once for each donor, with extended analysis performed for donor 1 (**a**). (**b**) Asterisk below p-IRF3 blot for donor 4 indicates a background band appearing below the true p-IRF3(S396) band, which was defined by ladder and detection of total IRF3 on a companion blot for all donors. N=4 experiments using 4 donors.

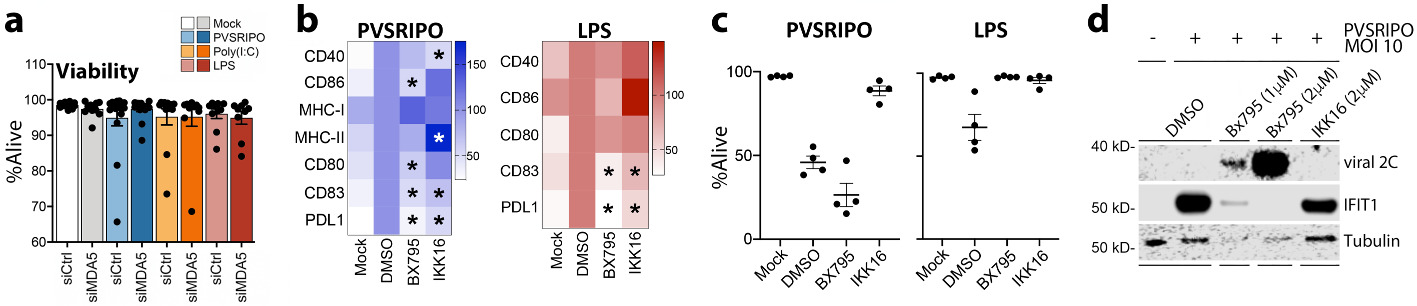

Supplementary Figure 9, related to Figure 6. Macrophage activation phenotype after PVSRIPO infection depends upon MDA5-TBK1 signaling.

(**a**) Cell viability by 7-AAD after control vs MDA5-targeting siRNA transfection, and stimulation with PVSRIPO/TLR agonists as shown in Fig. 6a-b (n=16 experiments PVSRIPO, n=10 LPS, n=12 all others; data bars represent mean -/+ SEM). (**b**) Heatmaps depicting levels of cell surface activation markers, and (**c**) viability (7-AAD) on cells from experiments in Fig. 6c (n=4 experiments; data brackets indicate mean -/+ SEM; (*) indicates Tukey's post-hoc p<0.05 vs DMSO treatment control. (**d**) Immunoblot analysis of viral protein (2C) and IFIT1 in the presence and absence of Bx795 or IKK16 during PVSRIPO infection, a representative series from n=2 experiments is shown.

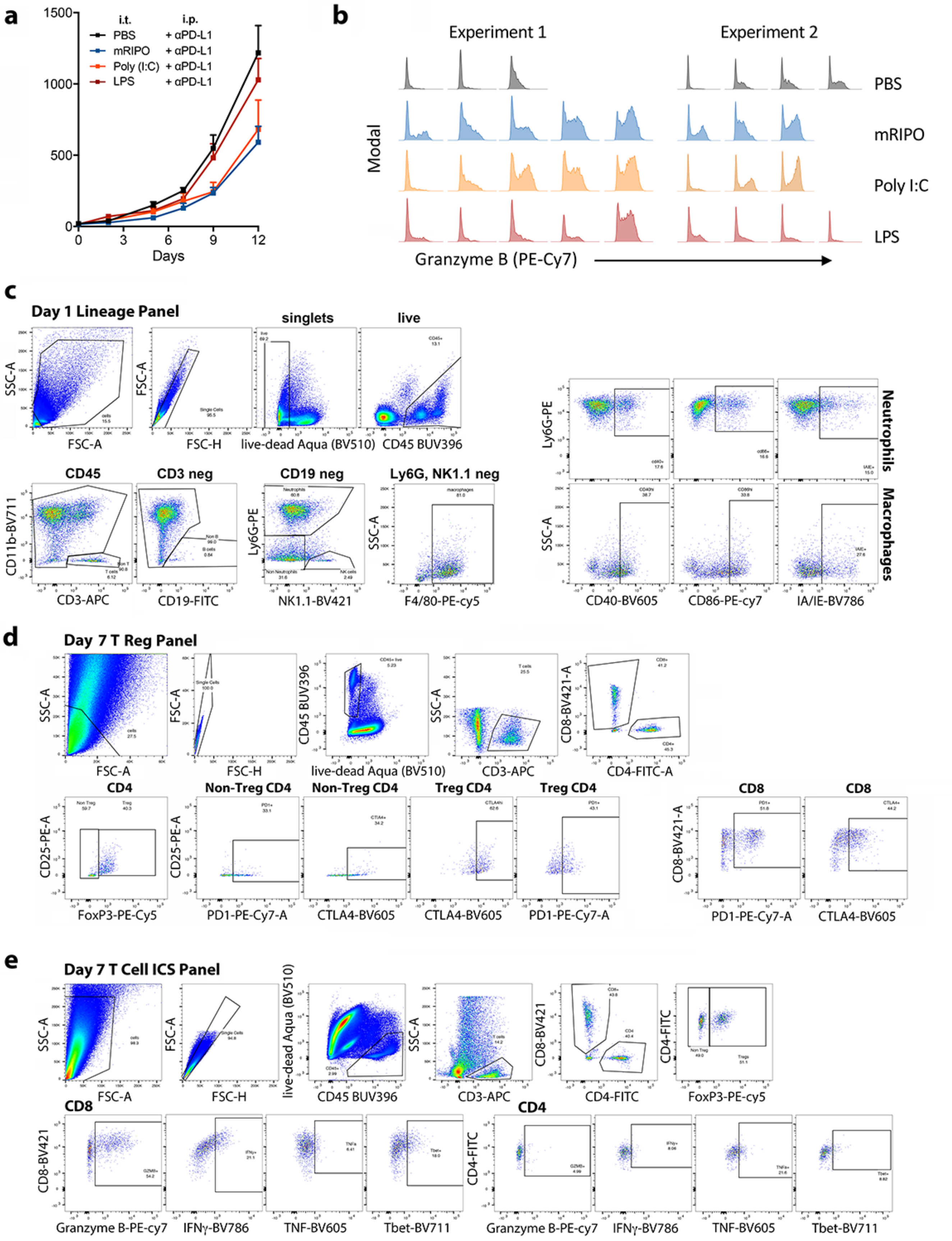
a
i.t.
i.p.
PBS + αPD-L1
mRIPO + αPD-L1
Poly (I:C) + αPD-L1
LPS + αPD-L1
Days
b
Experiment 1
Experiment 2
PBS
mRIPO
Poly I:C
LPS
Modal
Granzyme B (PE-Cy7)
c
Day 1 Lineage Panel
singlets
live
SSC-A
FSC-A
FSC-A
FSC-H
live-dead Aqua (BV510)
CD45 BUV396
CD45
CD3 neg
CD19 neg
Ly6G, NK1.1 neg
CD11b-BV711
CD3-APC
CD19-FITC
Ly6G-PE
NK1.1-BV421
SSC-A
F4/80-PE-cy5
Ly6G-PE
Neutrophils
SSC-A
Macrophages
CD40-BV605
CD86-PE-cy7
IA/IE-BV786
d
Day 7 T Reg Panel
SSC-A
FSC-A
FSC-A
FSC-H
CD45 BUV396
live-dead Aqua (BV510)
SSC-A
CD3-APC
CD8-BV421-A
CD4-FITC-A
CD4
Non-Treg CD4
Non-Treg CD4
Treg CD4
Treg CD4
CD8
CD8
CD25-PE-A
FoxP3-PE-Cy5
CD25-PE-A
PD1-PE-Cy7-A
CTLA4-BV605
CTLA4-BV605
PD1-PE-Cy7-A
CD8-BV421-A
PD1-PE-Cy7-A
CTLA4-BV605
e
Day 7 T Cell ICS Panel
SSC-A
FSC-A
FSC-A
FSC-H
live-dead Aqua (BV510)
CD45 BUV396
SSC-A
CD3-APC
CD8-BV421
CD4-FITC
CD4-FITC
FoxP3-PE-cy5
CD8
CD8-BV421
Granzyme B-PE-cy7
IFNγ-BV786
TNF-BV605
Tbet-BV711
CD4
CD4-FITC
Granzyme B-PE-cy7
IFNγ-BV786
TNF-BV605
Tbet-BV711

Supplementary Figure 10, related to Figure 7. mRIPO therapy is associated with polyfunctional tumor infiltrating T cell phenotypes.

(**a**) Repeat experiment for Figure 7a [n=5 for mRIPO/LPS, n=6 for PBS/Poly(I:C)]; mean tumor volume + SEM is plotted by treatment group. (**b**) Histograms of 2 experimental cohorts testing Granzyme B expression on CD8+ T cells after PBS, mRIPO, Poly(I:C) or LPS treatment, corresponding to data in Figure 7e. (**c**) Representative gating for macrophages, neutrophils, and NK cells along with relevant activation markers presented in Figure 7e; isotype control staining was used to define CD40, CD86, and IA/IE positivity. (**d**) Representative gating for Treg panel testing Treg, non-Treg, and CD8+ T cell expression of CTLA4 (intracellular stain), and PD1 in Figure 7e. (**e**) Representative gating for T cell panel assessing intracellular Granzyme B, IFNγ, TNF-α and T-bet expression by T cells as shown in Figure 7e; isotype control stained fluorescence minus one samples were used to determine positive intracellular staining (Granzyme B, IFNγ, TNF-α, and T-bet) populations.

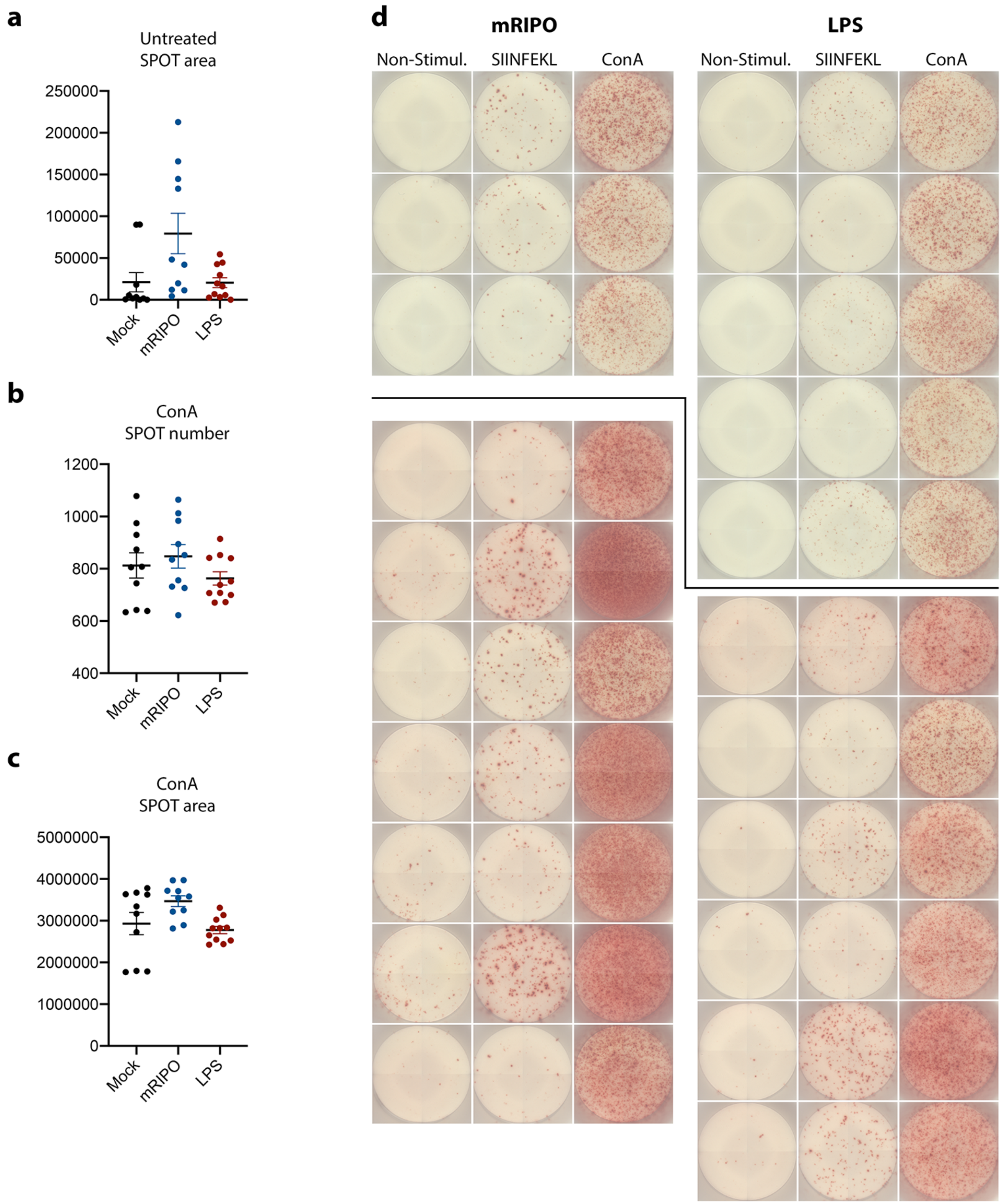
a
Untreated
SPOT area
250000
200000
150000
100000
50000
0
Mock
mRIPO
LPS
b
ConA
SPOT number
1200
1000
800
600
400
Mock
mRIPO
LPS
c
ConA
SPOT area
5000000
4000000
3000000
2000000
1000000
0
Mock
mRIPO
LPS
d
mRIPO
LPS
Non-Stimul.
SIINFEKL
ConA
Non-Stimul.
SIINFEKL
ConA

Supplementary Figure 11, related to Figure 8b. Additional analyses for ELISpot data presented in Figure 8b.

(**a**) Baseline total spot area for non-stimulated splenocytes. (**b**) Spot numbers for positive control (concanavalin A) treated splenocytes for ELISpot data presented in Figure 8b. (**c**) Total IFNγ spot area after Concanavalin A stimulation of splenocytes. (**a-c**) n=10 mice for PBS/mRIPO, n=11 mice LPS pooled from 2 experiments; data brackets indicate mean -/+ SEM. (**d**) Scans of ELISpot plates using splenocytes from tumor-bearing mice treated with mRIPO or LPS; the panels above and below the bar represent two independent experiments. In all ELISpot graphs/pictures all values for the mice with the 2 highest and 2 lowest IFNγ spot counts after SIINFEKL stimulation were excluded from each group to eliminate outliers; thus, the same samples (from the same mice) were excluded from all panels.

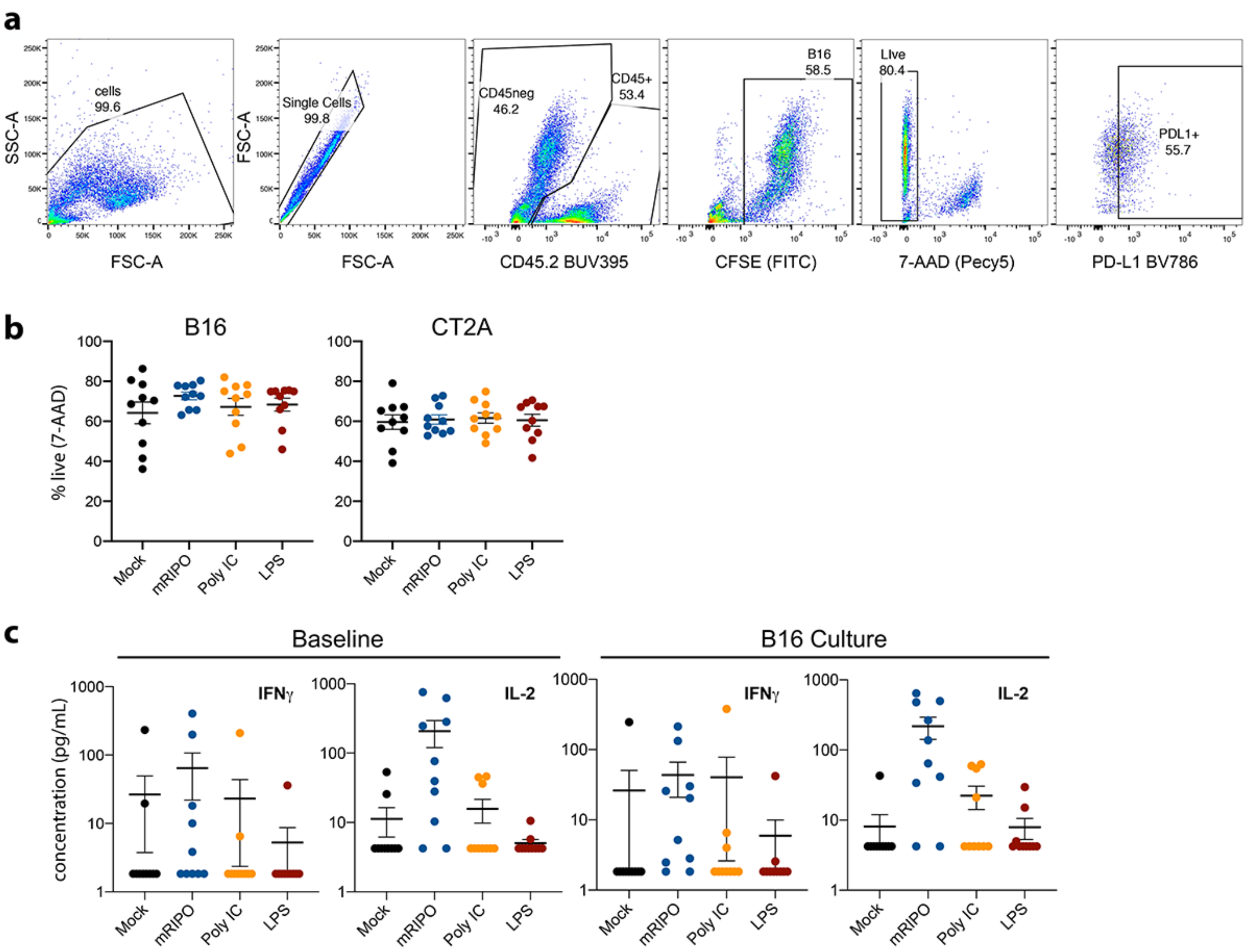

Supplementary Figure 12, related to Figure 8d. **Analysis of TDLN:B16 co-cultures seven days after mRIPO vs TLR agonist therapy.**

(**a**) Gating strategy for CFSE and PD-L1 analysis in Figure 8d. (**b**) %Live B16 and CT2A cells after TDLN cell co-culture presented in Figure 8d as measured by 7-AAD. (**c**) Supernatant cytokines were measured after 48h TDLN cell:B16 or CT2A co-culture from Figure 8d. (**b, c**) Data brackets represent mean -/+ SEM; n=10 mice per group.

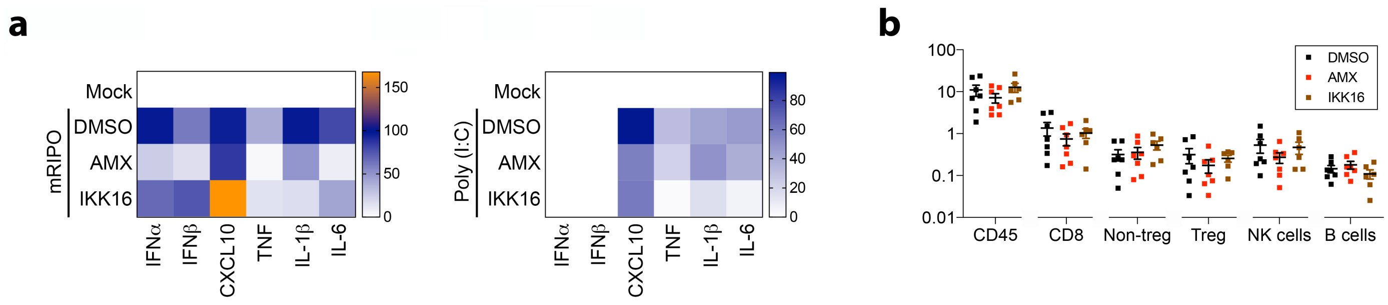

Supplementary Figure 13, related to Figure 9a, b. Confirmation of AMX inhibitor activity in murine cells and extended analysis of immunological changes after TBK1 inhibition.

(**a**) Heatmaps presenting cytokine release (24h) of DMSO, Amlexanox (2.5µM), or IKK16 (50nM) treatment of FLT3L-derived BMDCs in the presence of mRIPO (left, MOI 10) or Poly(I:C) (right, 10µg/ml). Percent maximum cytokine concentration (pg/ml) was determined, mock values were subtracted; mean normalized values are plotted [n=2 experiments]. (**b**) Extended analyses for tumor infiltrating cell type density (%live cells) for each indicated cell type for the experiment presented in Figure 9a. Data brackets represent mean -/+ SEM; n= 7/group mRIPO + DMSO and AMX, n=6 for mRIPO + IKK16.

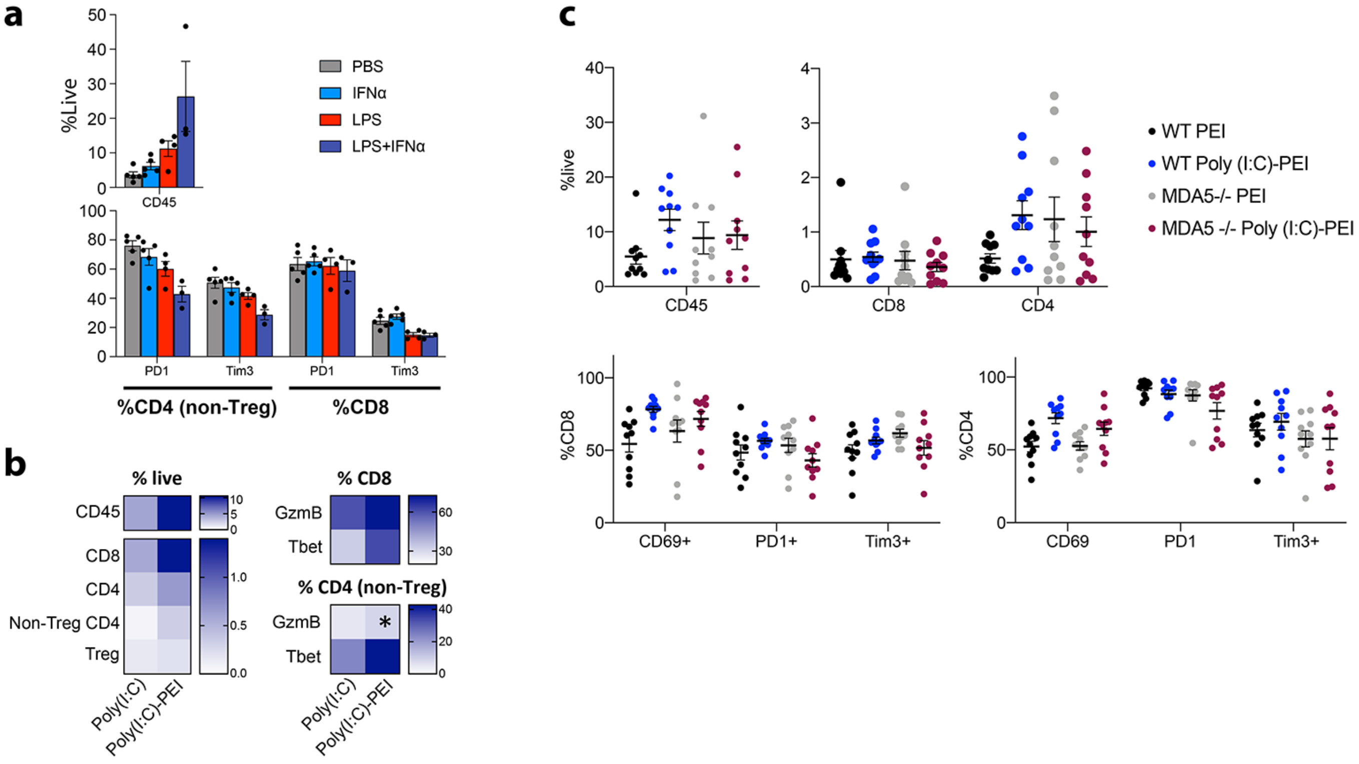

Supplementary Figure 14, related to Figure 9e, g, and i. Type-I IFN and cytoplasmic Poly(I:C) potentiate intratumor inflammation and T cell activation.

(**a**) Extended associated flow cytometry analyses for experiment performed in Figure 9e (n=3 mock/LPS + IFNα, n=5 IFNα, n=4 LPS only). (**b**) Tumors were harvested from surviving mice 16 days after treatment in the experiment described in Figure 9g and analyzed for immune infiltrates/T cell phenotypes [n=3 Poly(I:C), n=7 Poly(I:C)-PEI]; (*) t-test (two tailed) comparing Poly(I:C) vs Poly(I:C)-PEI, p=0.047**.** (**c**) Extended flow cytometry analyses for Figure 9i (n=10 mice/group). (**a, c**) All brackets/data bars represent mean -/+ SEM.
