# Supplementary material for: Viral infection of cells within the tumor microenvironment mediates antitumor immunotherapy via selective TBK1-IRF3 signaling
Source: Nat Commun. 2021 Mar 25;12:1858. doi: 10.1038/s41467-021-22088-1 (PMC7994570; doi:10.1038/s41467-021-22088-1)
Supplement: Supplementary file 3 — Reporting Summary [file 41467_2021_22088_MOESM3_ESM.pdf]

nature research

Corresponding author(s): Matthias Gromeier

Last updated by author(s): Jan 21, 2021

Reporting Summary

Nature Research wishes to improve the reproducibility of the work that we publish. This form provides structure for consistency and transparency in reporting. For further information on Nature Research policies, see our Editorial Policies and the Editorial Policy Checklist.

Statistics

For all statistical analyses, confirm that the following items are present in the figure legend, table legend, main text, or Methods section.

n/a	Confirmed	
☐	☒	The exact sample size (*n*) for each experimental group/condition, given as a discrete number and unit of measurement
☐	☒	A statement on whether measurements were taken from distinct samples or whether the same sample was measured repeatedly
☐	☒	The statistical test(s) used AND whether they are one- or two-sided<br>*Only common tests should be described solely by name; describe more complex techniques in the Methods section.*
☐	☒	A description of all covariates tested
☐	☒	A description of any assumptions or corrections, such as tests of normality and adjustment for multiple comparisons
☐	☒	A full description of the statistical parameters including central tendency (e.g. means) or other basic estimates (e.g. regression coefficient) AND variation (e.g. standard deviation) or associated estimates of uncertainty (e.g. confidence intervals)
☐	☒	For null hypothesis testing, the test statistic (e.g. *F*, *t*, *r*) with confidence intervals, effect sizes, degrees of freedom and *P* value noted<br>*Give P values as exact values whenever suitable.*
☒	☐	For Bayesian analysis, information on the choice of priors and Markov chain Monte Carlo settings
☒	☐	For hierarchical and complex designs, identification of the appropriate level for tests and full reporting of outcomes
☒	☐	Estimates of effect sizes (e.g. Cohen's *d*, Pearson's *r*), indicating how they were calculated

Our web collection on statistics for biologists contains articles on many of the points above.

Software and code

Policy information about availability of computer code

Data collection	BD FACs Diva software was used to collect flow cytometry data from the FortessaX20. Image Studio v5.2 was used for capturing immunoblot data using a Licor Odyssey Fc2.
Data analysis	Graphpad Prism v8 was used for statistical analyses, Legendplex v8 (Biolegend) software was used to determine concentration of cytokines after Legendplex assays, Flowjo v10 (BD biosciences) was used for analysis of flow all cytometry data, BioVinici v1.1.5 (Bioturing) was used for plotting of T-SNE plots.

For manuscripts utilizing custom algorithms or software that are central to the research but not yet described in published literature, software must be made available to editors and reviewers. We strongly encourage code deposition in a community repository (e.g. GitHub). See the Nature Research guidelines for submitting code & software for further information.

Data

Policy information about availability of data

All manuscripts must include a data availability statement. This statement should provide the following information, where applicable:

- Accession codes, unique identifiers, or web links for publicly available datasets
- A list of figures that have associated raw data
- A description of any restrictions on data availability

Source data are provided with this paper. All raw data used to construct figures in this study are included in the source data file.

Field-specific reporting

Please select the one below that is the best fit for your research. If you are not sure, read the appropriate sections before making your selection.

- [x] Life sciences
- [ ] Behavioural & social sciences
- [ ] Ecological, evolutionary & environmental sciences

For a reference copy of the document with all sections, see nature.com/documents/nr-reporting-summary-flat.pdf

Life sciences study design

All studies must disclose on these points even when the disclosure is negative.

Sample size

For glioma, breast, and melanoma ex vivo tumor slice assays the maximum number of surgical resection tissue cases available to our group for these studies from Oct 2016 to Sept 2019 were tested. Collected tumor tissue size determined whether sufficient (generally ~1cm in diameter or more) tumor tissue was available for CD155 surface expression analysis (Fig 1b), viability and TAM phenotype analyses (Fig 1f, g), and analysis of single cell suspensions pre- and post- separation of CD14+ cells (Fig 1h). For in vitro datasets using PBMC derived cells (MDMs, DCs) sample size was determined by treatment effect and standard error from two pilot experiments; PBMCs from multiple leukopak donors was used. For mouse studies testing mRIPO therapy, data from prior studies indicated a minimum of 6-7 mice per group for statistically significant differences in tumor growth after treatment. For flow cytometry analyses of tumor infiltrating immune cells, the sample size was chosen based upon a previously published analysis by our group analyzing neutrophil, T cell, and myeloid cell infiltration after mRIPO therapy (Brown et al STM 2017) and an initial pilot study using the flow cytometry panels performed in this study. For other in vivo studies a group size of 7-10 mice were chosen based upon pilot study treatment effects and/or published treatment effects for the tested drugs (e.g. Poly I:C, LPS, IFN, etc.). All experiments were performed multiple times.

Data exclusions

In Fig 1c & d, and Fig 1h one sample from each were excluded prior to testing due to visible contamination in the culture. In some mouse tumor growth studies euthanasia was required (per the IACUC protocol) prior to completed tumor growth time course because of tumor ulceration. For mice meeting the endpoint of 1000mm^3 tumor volume, the end-point value of '1000' was carried forward as a place holder for subsequent time points. Equal range and distribution of tumor volume between groups prior to treatment was a pre-established criteria for all mouse tumor studies to ensure valid average tumor volume comparisons. Thus, mice with tumor volumes of >2x the standard deviation from the mean (pre-established threshold) at pre-treatment/initial measurement were excluded by removing the equivalent number of largest and/or smallest tumors from each group in Figures 2b and d, 3e, 7a, 8f and 9d. Similar mean -/+ SEM and range between different treatment groups was confirmed for all intratumor treatment studies at the pre-treatment time point. In Fig 7e, one ulcerated tumor ~2x smaller than all others at time of harvest was excluded due to concerns of normalizing immune cell density to total live cell values; indeed outlier (>2x stdev from the mean) differences in calculated immune cell density from flow cytometry data from this sample was observed. For analyses in Fig 8b-d largest and smallest values were removed from each treatment group to exclude outliers defined by 2x stdev from the mean. Exclusion of outliers using >2x stdev from the mean within a given treatment group was a pre-established exclusion criteria for these studies (Fig 7e and 8b-d). In Fig 7e one specimen in the mRIPO group was not analyzed for CD69 or Tim3 (one of the panels was not recorded for this sample) due to data acquisition error.

Replication

All repeats of experiments were successful in replicating findings presented here. Group size, mean -/+ SEM, repeat experiment information, and statistical analyses are presented in figure legends. Representative experiments were chosen for presentation in figures, and in some cases repeat experiments are shown in the data supplement.

Randomization

All mouse experiments were randomized by cage; equal male and female mice were included in each group for B16 studies, in E0771 and MDA5-/- vs WT C57BL6 studies only female mice were used. For in vitro PBMC experiments different treatments were tested in the same preparations of PBMCs/macrophages/DCs for each figure, in general testing material from at least two de-identified leukopak donors in multiple experiments as indicated in figure legends. For ex vivo slice culture experiments, all available tissue was tested, and tissue was initially dissected into quarters followed by further dissection and allocation to culture vessels such that at least one slice from each tumor quarter was present in each treatment condition; slices were otherwise randomly allocated into culture vessels that were randomized to treatment groups after slice allocation.

Blinding

Investigators were blinded to treatment group allocation during: all tumor volume measurements, all flow cytometry data acquisition and analysis in Figures 7-9, ELISpot data acquisition and analysis (Figure 8b), and cytokine data acquisition and analysis in Figures 7c and 8c. Other in vitro-based experiments were not deemed necessary or feasible to perform blinded due to the availability of internal, sample-intrinsic controls relevant to the comparisons reported in this study (e.g. CD155 expression on different cell types in the same stained sample, TNF/IL-6 vs type I/III IFN in cytokine assessments on the same multivalent cytokine bead array preparations; CD40 vs CD80 expression in flow analyses), automated data analyses (e.g. legendplex software was used to auto-gate and analyze cytokine data), and limited manpower to blind smaller experiments that were repeated several times. Blinding was not feasible for immunoblots due to the need for the investigator to link (and annotate) sample lane location and sample identity during data acquisition.

Reporting for specific materials, systems and methods

We require information from authors about some types of materials, experimental systems and methods used in many studies. Here, indicate whether each material, system or method listed is relevant to your study. If you are not sure if a list item applies to your research, read the appropriate section before selecting a response.

Materials & experimental systems

n/a	Involved in the study	
	x	Antibodies
	x	Eukaryotic cell lines
x		Palaeontology and archaeology
	x	Animals and other organisms
	x	Human research participants
x		Clinical data
x		Dual use research of concern

Methods

n/a	Involved in the study	
x		ChIP-seq
	x	Flow cytometry
x		MRI-based neuroimaging

Antibodies

Antibodies used

Human antibodies:
FcX Trustain 422302 Biolegend
CD45-BUV395 563792 BD biosciences
CD14-BV421 325628 Biolegend
CD33-BV510 303422 Biolegend
CD49d-BV605 304324 Biolegend
HLA-DR-BV785 307642 Biolegend
CD31-FITC 303104 Biolegend
CD3- BUV737 612750 Biolegend
CD19-BUV737 564303 Biolegend
CD11b-APC 982604 Biolegend
CD16-BV711 302044 Biolegend
CD15-APC-fire7 323042 Biolegend
CD155-PE 337610 Biolegend
isptype-PE 400114 Biolegend
CD11b-Alexa Fluor 488 301318 Biolegend
CD40-APC 334309 Biolegend
CD86-BV510 305432 Biolegend
PD-L1-BV605 329724 Biolegend
CCR7-PE 353204 Biolegend
CD83-BV711 305334 Biolegend
CD80- BV421 305222 Biolegend
HLA-A, B, C-FITC 311404 Biolegend
Isotype APC 400120 Biolegend
isotype BV421 400158 Biolegend
isotpye BV510 400346 Biolegend
Isotype BV605 400350 Biolegend
Isotype BV786 400274 Biolegend
isotype BV711 400168 Biolegend
K1 anti-dsRNA antibody 10020500 Scicons
goat anti mouse IgG-APC 405308 Biolegend
donkey anti-rabbit IgG-Alexa Fluor 594 406418 Thermofisher
p-IRF3(S396) 4947 Cell Signaling Technologies
IRF3 11904 Cell Signaling Technologies
p-P65 NFkB(S536) 3033 Cell Signaling Technologies
p65 NFkB 8242 Cell Signaling Technologies
IRF7 4920 Cell Signaling Technologies
IRF1 8478 Cell Signaling Technologies
P50/105 NFkB 13586 Cell Signaling Technologies
P52/P100 NFkB 4882 Cell Signaling Technologies
A20 5630 Cell Signaling Technologies
c-REL 4727 Cell Signaling Technologies
IkBa 4812 Cell Signaling Technologies
IFIT1 14769 Cell Signaling Technologies
PKR 2766 Cell Signaling Technologies
CD155 13544 Cell Signaling Technologies
OAS1 14498 Cell Signaling Technologies
p-STAT1(Y701) 9167 Cell Signaling Technologies
STAT1 9172 Cell Signaling Technologies
TAP1 12341 Cell Signaling Technologies
p-p38 4511 Cell Signaling Technologies
p38 9212 Cell Signaling Technologies

PPARg 2435 Cell Signaling Technologies
Acetyl-coA Carboxylase 3676 Cell Signaling Technologies
IDO 12006 Cell Signaling Technologies
ISG15 2758 Cell Signaling Technologies
MDA5 5321 Cell Signaling Technologies
Tubulin T5168 Millipore-Sigma
poliovirus 2C a gift of E. Wimmer, Stony Brook Univ., NY

Mouse antibodies
CD40-BV605 745218 BD Biosciences
CD86-PEcy7 105014 Biolegend
PDL1-APC 124312 Biolegend
I-A/I-E-BV786 107645 Biolegend
H2Kb-PE 116508 Biolegend
isotype PE 400212 Biolegend
isotype BV605 400540 Biolegend
isotype PE-cy7 400522 Biolegend
Iostype BV786 400647 Biolegend
CD11c-APC 117310 Biolegend
CD45-BUV395 564616 BD Biosciences
CD11b-BV711 101242 Biolegend
F4/80-PECy5 123112 Biolegend
Ly6G-PE 127608 Biolegend
NK1.1-BV421 108732 Biolegend
CD3-FITC 11-0032-82 Thermo-Fisher
CD19-FITC 152404 Biolegend
CD3-APC 100236 Biolegend
CD4-PE 100512 Biolegend
CD8-BV421 101242 Biolegend
CD69-BV605 104530 Biolegend
CD44-PECy5 103010 Biolegend
CD62L-BV786 104440 Biolegend
TIM3-BV711 119727 Biolegend
PD1-PECy7 25-9985-82 Thermo-Fisher
CTLA4-BV605 106323 Biolegend
CD4-FITC 100510 Biolegend
FoxP3-PEcy5 15-5773-82 Thermo-Fisher
CD25-PE 12-0251-81A Thermo-Fisher
isotype PE/cy5 15-4321-80A Thermo-Fisher
Granzyme B-PECy7 372214 Biolegend
IFNg-BV786 505838 Biolegend
TNF-a-BV605 502936 Biolegend
Tbet-BV711 644820 Biolegend
isotype BV711 400168 Biolegend
CD11c-APC 117310 Biolegend
anti-IFNy capture ab (ELISpot) 517902 Biolegend
biotinylated anti IFN-y (ELISpot) 505704 Biolegend
anti-PD-L1 (therapy antibody) BE0101 BioXcell
anti-IFNAR (therapy antibody, MAR1-5A3) BE0241 BioXcell
mouse IgG1 control BE0083 BioXcell

Validation

All antibodies used for flow cytometry analyses were previously validated by the manufacturer using human PBMCs or mouse splenocytes vs isotype control staining comparing cell types/conditions known to be positive and negative for the relevant antigens; these data are shown on the manufacturer's websites. We confirmed the specificity of antibodies via comparison of antibody staining on cell types known to be negative (e.g. CD8 T cells did not express FoxP3, T cells did not express F4/80), cell types known to be positive (e.g. a subset of CD4 T cells expressed FoxP3, macrophages expressed F4/80); and comparison to cells stained with isotype control antibody. The specificity of mouse specific antibodies used to measure T cell activation/exhuastion phenotype (e.g. Granzyme B, Tbet, IFN-y, TNF, CD69, PD1, TIM3) was also confirmed by staining OT-I cells with and without stimulation with SIINFEKL peptide (for 48h). The specificity of macrophage/DC activation markers tested by flow cytometry was confirmed by comparing macrophages pre- and post- stimulation with innate PRR agonists in this study (for both mouse and human specific antibodies). Antibodies used for immunoblot were validated by immunoblot on known positive and negative cells/treatment conditions by the manufacturer. The specificity of the antibodies for immunoblot was confirmed using predicted size (KDa) via comparison to protein ladder, as well as confirming established behavior of the recognized antigen after treatment with PRR activators (e.g. IRF3 phosphorylation, ISG expression, and NFkB signaling are induced after viral infection/PRR signaling). We did not test/confirm species specificity of antibodies, as distinguishing between different species was not an objective/requirement of this study. For antibodies used for in vivo functional studies, anti-PDL1 and anti-IFNAR, the manufacturer (BioXcell) confirmed that these antibodies block PDL1 and IFNAR signaling, respectively, and bind recognize PDL1 and IFNAR via immunoblot, this information is available on the manufacturer's website. In addition, both clones have been used extensively by several groups to block PDL1 and IFNAR signaling in vivo and in vitro. The negative control antibody (mouse IgG1 control) has no known specificity, but the antibody clone (MOPC-2) is commonly used as an isotype control in functional studies. The antibodies used for ELISpot were validated for this purpose by the manufacterers, and

was validated by our group by using positive and negative control conditions to measure IFN-y secretion, and positive (ConA) and negative control (unstimulated) signal confirmed specificity to activated T cells.

Eukaryotic cell lines

Policy information about cell lines

Cell line source(s)	HeLa R19 (Dr E. Wimmer, State Univ, of NY at Stony Brook)<br>B16-F10 (ATCC)<br>B16-F10.9-OVA (Dr. S. Nair, Duke University)<br>B16-F10.9-OVA-CD155 (Previously validated in Brown et al STM 2017)<br>B16-F10-CD155 (generated in this study as described in the methods)<br>E0771 (Dr. G. Palmer, Duke University)<br>CT2A (Dr. P Fecci, Duke University)<br>U87 (ATCC)<br>Du54, Du43 (Dr. D. Bigner, Duke University)
Authentication	OVA expression was confirmed via OT-I assay in the B16F10.9OVA cell line. For CD155 transgenic lines, CD155 expression was confirmed by flow cytometry and by susceptibility to lysis by mRIPO. Melanin expression was confirmed in all B16 cell lines by visible inspection upon cell preparation for tumor implantation. C57BL/6 origin of CT2A was confirmed by whole exome sequencing. HeLa R19, E0771, Du54, U87, and Du43 cell lines were not independently authenticated by our group.
Mycoplasma contamination	All cell lines tested negative for mycoplasma at the beginning of, and several times throughout, the study.
Commonly misidentified lines<br>(See ICLAC register)	None

Animals and other organisms

Policy information about studies involving animals; ARRIVE guidelines recommended for reporting animal research

Laboratory animals	C57BL/6J mice (strain code 000664) and MDA5 -/- (strain code 015812) were purchased from Jackson Labs. CD155-tg C57BL/6 mice were a generous gift of Satoshi Koike (Tokyo Metropolitan Institute of Medical Science, Japan). Six- to ten-week-old male and female mice were used for B16 studies, with equal distribution of males and females in each treatment group; female mice were used for E0771 studies (8 - 10 weeks old) and MDA5-/- vs WT comparison studies (6 - 8 weeks old) in B16. Mice were housed in the Duke University Cancer Center Isolation Facility with 12 hours light/12 hours dark cycles, a relative humidity of 50 -/+ 20%, and temperature of 21 -/+ 3oC.
Wild animals	No wild animals were used in this study
Field-collected samples	No field collected samples were used in this study
Ethics oversight	All mouse experiments were performed under an approved Duke University IACUC protocol.

Note that full information on the approval of the study protocol must also be provided in the manuscript.

Human research participants

Policy information about studies involving human research participants

Population characteristics	Patients with glioblastoma, melanoma, or breast cancer undergoing surgical resection.
Recruitment	Cancer patients were consented for de-identified tissue collection for use in research, only diagnosis/tumor-type was provided to the researchers. Pathologically confirmed GBM tumor tissue was collected within 1h of resection by the Duke Preston Robert Tisch Brain Tumor Center BioRepository (PRTBTBR); de-identified breast cancer and melanoma tumor tissues (of varying grade and subtype) were collected by the Duke Bio-repository and Precision Pathological Center (BRPC). The collection of these samples may be biased towards larger tumor size at time of surgery, as excess tissue needed for pathology and other ongoing research studies was used in this study. Otherwise, the accrual of tumor tissue in this study is anticipated to match the distribution of patients with glioblastoma, melanoma, and breast cancer at Duke University undergoing surgical resection. This is not anticipated to impact the results or conclusions of our study, but may bias the tissues tested towards certain baseline features (e.g. necrosis, aggressive growth, immunosuppression).
Ethics oversight	The Duke University Institutional Review Board (IRB) reviewed and approved this work via study associated protocol approval.

Note that full information on the approval of the study protocol must also be provided in the manuscript.

Flow Cytometry

Plots

Confirm that:

- [x] The axis labels state the marker and fluorochrome used (e.g. CD4-FITC).
- [x] The axis scales are clearly visible. Include numbers along axes only for bottom left plot of group (a 'group' is an analysis of identical markers).
- [x] All plots are contour plots with outliers or pseudocolor plots.
- [x] A numerical value for number of cells or percentage (with statistics) is provided.

Methodology

Sample preparation	For tumor specimens cells were dissociated (liberase and DNAse I) as described in methods, washed in PBS, stained with Zombie Aqua, washed in FACs buffer (2% FBS+ PBS), Fc-blocked, and stained with surface antibodies. For surface stains only, cells were stained for 1hr and washed in FACs buffer, filtered through a 70um cell strainer, followed by reconstitution in fresh FACs buffer for analysis. If intracellular staining was performed, the FoxP3 staining kit or the intracellular fixation and permeabilization kit (both Thermo Fisher) instructions were used. For mouse and human cells not derived from tumors, the same protocols were used, with exception of dissociation. In some cases 7-AAD was used instead of Zombie Aqua, and was added to the FACs buffer used to re-dissociate pellets after the last wash (only after surface staining protocols).
Instrument	BD Fortessa X 20 (Duke Cancer Institute Flow Cytometry Core Facility).
Software	BD FACs DIVA for acquisition; Flow Jo v10 was used for compensation and analysis.
Cell population abundance	For analyses of tumor infiltrating immune cells a minimum of 1x10^5 live singlets were analyzed, and a minimum of 400 T cells were analyze. For analysis of monocyte derived/mouse PEC/BMDCs a minimum of 1000 events were recorded.
Gating strategy	In general: FSC-A vs SSC-A was used to gate a total cell population, FSC-H (or SSC-H) vs FSC-A (or SSC-A) were used to excluded doublets, and live cells were selected after staining for dead cells (either via Zombie Aqua or 7-AAD). Gating for cell types followed by markers on cell types of interested were performed thereafter. Gating strategies for each select panel are presented in supplementary figures.

- [x] Tick this box to confirm that a figure exemplifying the gating strategy is provided in the Supplementary Information.
